# A Hybrid Adaptive Routing Algorithm for Event-Driven Wireless Sensor Networks

**DOI:** 10.3390/s90907287

**Published:** 2009-09-10

**Authors:** Carlos M. S. Figueiredo, Eduardo F. Nakamura, Antonio A. F. Loureiro

**Affiliations:** 1 Analysis, Research and Technological Innovation Center, Av. Gov. Danilo de Matos Areosa, 381, Distrito Industrial, 69075-351, Manaus, AM, Brazil; E-Mail: eduardo.nakamura@fucapi.br; 2 Department of Computer Science, Federal University of Minas Gerais, Av. Antonio Carlos, 6627, 31270-010, Belo Horizonte, MG, Brazil; E-Mail: loureiro@dcc.ufmg.br

**Keywords:** wireless sensor networks, self-adaptive routing, self organization, proactive routing, reactive routing, moving average, estimation

## Abstract

Routing is a basic function in wireless sensor networks (WSNs). For these networks, routing algorithms depend on the characteristics of the applications and, consequently, there is no self-contained algorithm suitable for every case. In some scenarios, the network behavior (traffic load) may vary a lot, such as an event-driven application, favoring different algorithms at different instants. This work presents a hybrid and adaptive algorithm for routing in WSNs, called Multi-MAF, that adapts its behavior autonomously in response to the variation of network conditions. In particular, the proposed algorithm applies both reactive and proactive strategies for routing infrastructure creation, and uses an event-detection estimation model to change between the strategies and save energy. To show the advantages of the proposed approach, it is evaluated through simulations. Comparisons with independent reactive and proactive algorithms show improvements on energy consumption.

## 1. Introduction

A Wireless Sensor Network (WSN) [1, 2] is a special type of ad hoc network that has become popular due to its wide applicability, which includes environmental, medical, industrial, and military applications [3]. A WSN comprises a large number of low-cost sensor nodes that can perceive the environment and perform collaborative tasks to monitor an area of interest.

Because WSNs are resource-constrained networks—regarding energy, bandwidth, and computational capacity—algorithms and solutions for WSNs need to be resource efficient. In addition, WSNs demand self-organizing features, i.e., the ability of autonomously adapt to changes resulting from external interventions, such as topological changes (cause by failures, mobility, or node inclusion) or reaction to an event detection.

An important issue in WSNs is collecting and processing data perceived from the environment and sending that data to be processed and evaluated by an external entity connected to a sink node (or gateway). As a consequence, routing data towards the sink node is a fundamental task in WSNs. Due to the resource constraints, different algorithms have been proposed considering specific application and scenario characteristics [4]. Thus, given a specific scenario, a WSN may be designed to operate with the most suitable routing algorithm, which can be defined *a priori*.

Regarding the application characteristics, WSNs can demand different data collecting strategies, such as periodic, eventual, or on-demand strategies [5]. In particular, we expect that several applications should adopt the event-driven model. These applications are concerned with special events that should not be so frequent (e.g., fire or intrusion detection), so that the network must work in a low activity mode until an event occurs. In such scenarios, the network behavior may frequently change in an unpredictable way. For example, the network may present a low traffic profile for a long time, but after a while a few events occur, which increase the traffic load and the routing workload as well. Such a feature favors different algorithms in different situations. However, it might be unfeasible or undesirable to have an external entity dynamically acting on the network to change its behavior. In this case, the network must adapt itself based on autonomic principles.

Hybrid adaptive approaches for routing are natural choices to deal with dynamic scenarios by choosing the best routing strategy in response to current network conditions. In this context, some protocols have been proposed for ad hoc networks (MANETs), such as ZRP [6] and SHARP [7]. However, they are not suitable for WSNs due to fundamental differences in the network, applications, and traffic profiles. The first effort in the WSN domain was made by us in [8], which initially proposed a hybrid solution composed of traditional reactive and proactive routing strategies for WSNs by using a simple traffic limiar, and in [9], which improves the adaptation rule by considering the event occurrence characteristic. In fact, this article is an extended version of these work.

In this work, we evolve this approach by considering the temporal characteristic of event occurrence and detection through an estimation model. Thus, the proposed solution, called Multi-MAF, captures the seasonal and dynamic characteristics of events for predicting and choosing which routing strategy should be used: a reactive or proactive strategy. The Multi-MAF algorithm is evaluated through a set of simulations that compares the hybrid approach to their reactive and proactive components, and other well-known proposals.

The remainder of this paper is organized as follows. Section 2 presents a broader discussion about data routing in WSNs. Section 3 presents the Multi-MAF algorithm by describing its reactive and proactive components and the adopted adaptation model. Section 4 evaluates Multi-MAF by comparing the hybrid solution to its individual reactive and proactive components. Finally, Section 5 presents our final considerations and outlook.

## 2. Related Work

The routing task in WSNs differs from traditional (and ad hoc) networks in many aspects. In WSNs, energy efficiency is a major concern, since sensor nodes are battery powered. In addition, a common task in a WSN is to collect and process data acquired from the environment and send it to be further processed and evaluated by an external entity connected to a sink node (or gateway). Hence, routing data towards the sink node is a fundamental task and different algorithms have been proposed [4] for different cases or scenarios due to their particularities.

Routing protocols may be classified according to different criteria. Considering the strategy for infrastructure creation and maintenance, we have three major classes:

**Flooding and Gossiping** [10]. These are classical mechanisms to forward data in sensor networks that do not need to maintain any routing infrastructure or topology. In the flooding algorithm, each data is iteratively broadcasted to all neighbors until the destination is reached. Gossiping differs from flooding by choosing random nodes to forward the data. Although this approach avoids the cost of creating and maintaining routes, it causes the data packet implosion problem which represents an excessive cost for WSNs.

**Proactive protocols**. In this class of protocols, routing infrastructure is created and constantly maintained, independent of network behavior. In general, this process is performed by destination nodes. Examples of this approach are DSDV [11] for MANETs and various tree-based protocols [12] for WSNs (e.g., One-Phase Pull Diffusion [13]). This approach can result in improved routing, but has the disadvantage of constant resource consumption. Recent contributions like BVR [14], VRR [15] and S4 [16] are interesting evolutions of classical routing approaches. They all present proactive behavior due to the need of setting and updating routing information, and they present improvements regarding routing metrics and state information decreasing, an important feature for practical scalable WSNs.

**Reactive protocols**. In this class, the routing infrastructure is created only when a node needs to transmit a packet. AODV [17] is a well-known protocol for MANETs, and Push Diffusion [13], InFRA [18] and LRDE [19] are examples of reactive protocols for WSNs. Such an approach saves resources during inactivity periods, but has the overhead for discovering paths for every originator node.

**Hybrid protocols**. Another class is composed of hybrid adaptive algorithms, which choose a reactive or a proactive strategy based on network conditions. Some hybrid protocols have been proposed for MANETs. The ZRP protocol (Zone Routing Protocol) [6] is the first protocol to apply reactive and proactive strategies in a hybrid solution. The ZRP protocol delimits a zone around every node in such a way that routes are proactively updated inside the zones, and reactively outside the zones. The main goal of the ZRP protocol is to reduce the routing overhead. The SHARP protocol [7] extends this approach in such a way that zones can be dynamically determined only around nodes with significant incoming data, and it also allows adaptation with other application-specific metrics, such as jitter and loss rate, in addition to routing overhead.

Such a hybrid adaptive approach has not been widely adopted in WSNs. In addition, current MANET protocols are not suitable for sensor networks due to several particularities inherent in such resource-constrained networks. Unlike MANETs, in which communication is essentially many-to-many, in WSNs communication is usually many-to-one, since data flow from sources to a sink node or from sources to a cluster-head. This characteristic provides a broader view for a sink or cluster-head to perform an adaptive control of the nodes under its responsibility. The traffic characteristic also differs between these networks, and this fact must be considered in an adaptive model. While in MANETs the nodes can dictate how data is generated according to their independent applications and user needs, in WSNs this can be dictated by an external application for the whole network according to different requisition models or queries [5]. Also, for event-driven applications (and other in-network aggregation functions), we should consider spatial and temporal correlation among traffic of different nodes [20]. Again, all these characteristics can lead to a proper adaptive rule in the WSN domain, which is the goal of the Multi-MAF solution.

## 3. Multi-MAF: A Hybrid Adaptive Algorithm for Routing in WSNs

In this section, we provide a detailed discussion about the design principles of our hybrid routing algorithm for WSNs, which we call Multi-MAF.

### 3.1. An Overview

**Figure 1.**
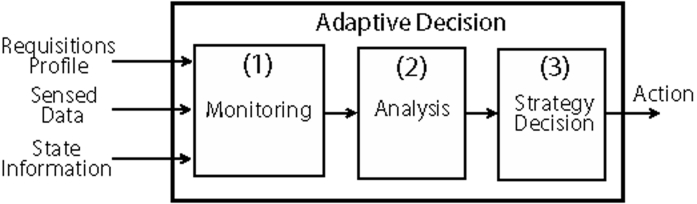
Adaptive Model.

An adaptive behavior refers to the network ability to react to some perceived situation. A generic adaptive model for data routing in WSNs is depicted in Figure 1. Here, a node (generally a sink or a cluster-head) is responsible for monitoring the network conditions (1), analyze its state (2), and decide what action (3) should be executed in a given moment.

The monitoring phase consists of receiving the sensed data and state information collected from the network. The profile of applications running on the network and the type of data request is also considered as a basis for decision making. The analysis may use accounting, threshold evaluation, prediction or inference techniques, depending on node resources and processing capacity. In this case, the goal is to detect situations in which some action is needed to achieve a better performance or to recover from an undesirable situation.

The Multi-MAF solution autonomously adapts its behavior in response to the variation of the network conditions according to the adaptive model described in Section 3.1. Multi-MAF is built using traditional reactive and proactive routing strategies for WSNs, and it applies an adaptive rule based on characteristics common to this kind of network. As a proof-of-concept, our implementation addresses particular algorithms and scenarios, but the insights presented here could guide the design of other adaptive hybrid solutions for WSNs.

The adaptive control of Multi-MAF is performed by the sink node that interacts with external applications and monitors the network traffic. Thus, many-to-one communication is assumed. Network clustering, a common approach in WSNs [21] to improve the network scalability, may be considered if the number of nodes and sensing area increases. The idea is that *cluster-heads* are responsible for coordinating the activities of all nodes in its coverage area, and, in our case, to perform the adaptive control.

Regarding the application’s requisition model, routing can be [5]: (1) continuous, (2) event-driven, or (3) observer-initiated. Proactive protocols are the natural choice for continuous and observer-initiated, because we know when the routing infrastructure will be necessary. However, with an event-driven model, we do not know when an event will occur. In this case, the network may be inactive or with a low activity for long periods (e.g., weeks or months). Thus, we should save energy during such inactive periods. In such cases, the reactive routing approach is preferable, because we do not have to proactively maintain the routing infrastructure, since it is created only when necessary. Even if we expect that sensor networks are not completely inactive for a long time (e.g., due to the need of periodical alive messages), we may not need to constantly update routes, which results in additional control messages and resource consumption. Alive status can be asked by the operator under longer periods of time, for example. However, at a given moment, several events might be detected generating a high traffic, which can be appropriated for proactive algorithms to avoid path discovery cost. In such cases, an adaptive model may be used to detect such conditions and choose the best routing strategy, saving energy.

### 3.2. Routing Mechanisms

The proactive and reactive components of Multi-MAF are classical approaches for WSNs, so they are not this paper’s contribution. Our goal is to show how a hybrid solution can be built having in mind the benefits of choosing a proactive or a reactive routing strategy, based on the current traffic profile. In addition, an integrated forwarding rule is also presented for hybrid operation.

#### Proactive Component: EF-Tree

**Figure 2.**
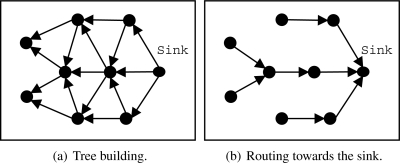
EF-Tree algorithm.

A simple and efficient structure for data dissemination is a routing tree [12], such as One-Phase Pull Diffusion [13]. Generally, the tree structure is created and maintained by a sink node that periodically rebuilds that tree to support eventual topological changes, link problems due to interference or traffic variations, node failures, etc. Our implementation, called EF-Tree (Earliest-First Tree) [22], is presented in Figure 2, which is described as follows:

- The sink node starts the process by broadcasting a building (control) message to all neighbors, as shown in Figure 2(a).
- When a node receives the building message, it sets the first sender as its parent and broadcasts the building message to all its neighbors, discarding building messages from other neighbors. Other parent selection strategies may be used, such as the node that belongs to the path of greater residual energy. An interesting practical approach is presented by Woo *et al.* [12], who used the radio link quality to choose the node’s parent. We intend to evaluate other parent selection strategies in future work.
- Whenever a node has a data packet to be transmitted (sensed or forwarded by another node), it sends that packet directly to its parent as shown in Figure 2(b).
- The building process is periodically repeated, so the network adapts itself to eventual topological changes, such as failures, node movements, and node additions. This periodicity depends on how frequent topological chances occur. The more dynamic the network, the shorter the rebuilding period.

#### Reactive Component: SID

On demand algorithms, such as AODV [17] and Push Diffusion [13], define another common approach for routing in ad hoc and sensor networks. In this strategy, the network may remain inactive until the communication process is triggered by sensors whose data is ready to be sent. This is the natural choice for event-driven scenarios in which communication may be triggered by an event detection. Obviously, the advantage of this approach is clearer in situations in which no management or control traffic is required, and periodical wake-up schemes, such as STEM [23] and B-MAC [24], can be used to save more resources while no event is being detected.

**Figure 3.**
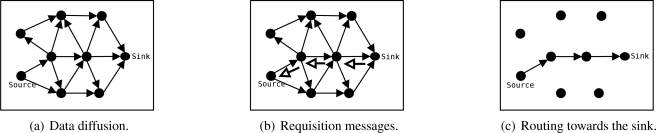
SID algorithm.

In this work, we propose a simple reactive algorithm, called SID (Source-Initiated Dissemination). SID is similar to the Push Diffusion [13]. However, SID allows source nodes to flood their data (e.g., when an event is detected) as long as a route is not available, and not only periodically as Push Diffusion proposes. These features make SID more reactive in the presence of network dynamics, but more sensitive in scenarios with intensive traffic. The SID protocol is decribed as follows:

- A source node that detects an event broadcasts the sensed data, its identification, and a timestamp to all neighbors [Figure 3(a)].
- Whenever a node receives the data sent by another node, it stores its data identification (source node identification and timestamp) and the sender’s identification. Eventually the node receives that packet from all neighbors, however, it will store and forward only the first packet. The amount of memory required by the algorithm is proportional to the network size. Other criteria for path formation may be considered, just as mentioned in the EF-Tree description.
- Similarly, data will arrive at the sink node from all neighbors. Then, the sink will send a control message requesting the data to be sent by the node from which it first received the data. This message identifies the data to be sent. This process is executed periodically by the sink while data is received.
- When a node receives this control message (data request), it repeats the process and identifies which node should send that data. This process is repeated until the source nodes are reached, as shown in Figure 3(b).
- Once a source node receives the control message requesting its data, it will update its own table so it will send further data packets to the node that first requested it. Thus, data is disseminated through the path where the sink’s requisition message arrived [Figure 3(c)], which might be the fastest path in symmetric channels.
- In order to allow the network to adjust to eventual topological changes (due to failures, mobility, or node inclusion), requisition messages are periodically sent by the sink towards the sources while data is being received. Once a node (source or relay) stops receiving requisition messages, due to any topological change, the node restarts to send or forward data by broadcast. Thus, if any path exists, data will reach the sink again and it will restart the requisition process.
- Once the events disappear, data traffic stop and, consequently, the sink node stops sending requisition messages to source nodes. In the absence of periodic requisition messages for a specific piece of data, the table entries expire and the network becomes inactive again.

#### Comparative Cost Analysis for EF-Tree and SID

In order to prove the situations in which each algorithm is preferable, we present a comparative cost analysis regarding energy consumption, which is the basis for the Multi-MAF adaptive model.

When a path is formed by SID and EF-Tree algorithms, we have an approximated energy cost (in Joules) of data dissemination given by:

(1) Cdata=Pt⋅Sd⋅Nh+Pr⋅Sd⋅Nh⋅Nn

representing the cost of data transmission by one source and routers, and the cost of signal reception by neighbors. *Pt* and *Pr* are transmission and reception power consumptions, respectively, in Joules/byte. *Sd* is the data size in bytes, *Nh* is the approximated number of hops from source to sink, and *Nn* is the approximated number of neighbors of a node.

The significant cost differences of the algorithms are due to the routing infrastructure formation. For the EF-Tree this energy cost is given by periodic broadcasts of tree construction messages, which is estimated by:

(2) Ctree=(Pt⋅Sc⋅N+Pr⋅Sc⋅Nn⋅N)⋅⌈(T/Ttree)⌉

where *Sc* is the control packet size (in bytes), *N* is the number of nodes in the network, *T* is the execution time and *Ttree* is the tree reconstruction period (both in seconds). The relation ⌈(*T/Ttree*)⌉ represents the number of tree buildings in the execution time.

SID does initial broadcasts for each source and the sink sends requisitions messages towards source nodes for path definition. In this case, we have:

(3) $$\begin{array}{lll} C\;\mathrm{sid} & = & \mathrm{Ns}\cdot (\mathrm{Pt}\cdot \mathrm{Sd}\cdot N+\mathrm{Pr}\cdot \mathrm{Sd}\cdot \mathrm{Nn}\cdot N+\\ & & +(\mathrm{Pt}\cdot \mathrm{Sc}\cdot \mathrm{Nh}+\mathrm{Pr}\cdot \mathrm{Sc}\cdot \mathrm{Nn}\cdot \mathrm{Nh})\cdot \left\lceil \left(\mathrm{Tev}/\mathrm{Tsid}\right)\right\rceil \end{array}$$

where *Ns* is the number of source nodes sending data, *Tev* and *Tsid* are, respectively, the average traffic duration for every source and the sink’s requisition messages interval (in seconds). The relation ⌈(*T/T sid*)⌉ gives the number of requisitions in the execution time.

**Proposition 1** *The cost of SID can exceed the cost of EF-Tree regarding the number of sources (Ns) in a time interval T.*

**Proof.** We can see that EF-Tree has a constant energy cost in function of the network lifetime (*T*). On the other hand, SID has an energy cost proportional to the number of sources (*Ns*). A crossing-point in the energy costs of the algorithms is expected to occur as the number of source nodes increases in a given execution time. This can be seen by making *Ctree* = *Csid*. For a simpler analysis, we can eliminate the second term of *Csid* by assuming that *Nh* (number of hops from source to sink) is much smaller than *N* (number of nodes), and *Tev* (event duration) tends to be much smaller than *T* (network lifetime). Also, we can consider a little difference between *Sd* and *Sc*, because *Sd* represents broadcasts for path discovery in *Csid* and, in practice, either this broadcast could be replaced by control packets (best case) or data packet are not much larger than control packets (*Sc* in *Ctree*), due to platform limitations (as it happens in Mica and is also considered in our simulation scenarios). Thus, the crossing point happens when the number of sources exceeds the number of tree-building in a given execution time.

Therefore, it is not appropriate to use the EF-Tree algorithm during inactive periods of traffic that are expected in event-driven applications, and the SID algorithm should be used when the event detection rate, which impacts in the number of sources, is low. These facts are explored on the proposed hybrid adaptive approach to achieve the best performance from both solutions.

#### Forwarding Rule

In a hybrid operation, the routing strategy chosen by the sink must be propagated to all network nodes and a broadcast control message might be used to switch the source nodes from one algorithm to another. In this work, we simplify this transition by assuming that all network nodes always respond normally to control messages as their original protocols determine, but validity is maintained through a timestamp and a predefined period of time given by the periodicity of the reconstructions and requisitions of EF-Tree and SID, respectively. Thus, we apply an integrated forwarding rule to automatically adjust the network according to the sink decision: every data packet (generated or forwarded) is sent through a routing tree if it is available (EF-Tree behavior); otherwise, data is sent to the neighbor from which a specific and valid requisition was received (SID behavior); otherwise, the data is sent by broadcast (Flooding).

#### Transition Analysis

Multi-MAF is composed of operating changes between a reactive and a proactive behavior controlled by the sink. In this section, we study what happens during these transitions and their impact on data routing behavior.

**Figure 4.**
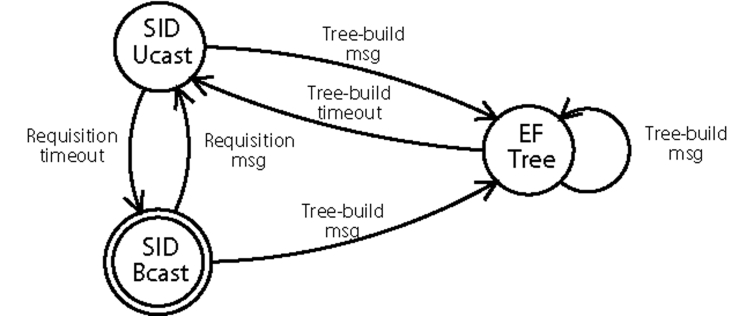
State diagram of Multi-MAF.

The initial routing state of Multi-MAF is SID and it changes to EF-Tree when a tree-build message is received. Multi-MAF stays in this state while periodical tree-build messages are received. A timeout leads the algorithm back to the SID state. Figure 4 shows the state diagram of Multi-MAF in which the SID state was divided into two operation modes for data forwarding: SID-Ucast and SID-Bcast for unicast and broadcast, respectively. During the time, different nodes in the network can be in different states. This may happen because: (1) The tree is under construction; (2) New nodes were added to the EF-Tree mode network; (3) The tree is not built for the entire network, due to packet losses; (4) The sink sends requisition messages, returning to the SID state, whereas part of the tree is still valid.

**Proposition 2** *Considering the proposed forwarding rule, the communication between nodes in different routing states (due to some of the causes described before) is possible.*

**Proof.** We show this characteristic in two parts:

From SID to EF-Tree state: Data generated or forwarded by a node in the SID-Ucast state can reach a node in the EF-Tree state, due to a tree construction for example (first case). Based on the forwarding rule described before, the packet will follow the tree path. If a data packet is flooded (SID-Bcast state), which occurs when the requisition message path is still not built, this packet can reach the tree in more than one point and it will be forwarded with duplications until it reaches a tree-node which has already received it. The last case is exactly what happens when new nodes are added to the network, because they always start in the SID-Bcast state.
From EF-Tree to SID state: This situation happens when the sink node decides to return to the SID mode, sending requisition messages to source nodes that may be sending their data through a still valid tree. As the SID requisitions follow the reverse path of the last received data, they will follow the paths of the tree being used for each source. When the tree becomes invalid in a node which already received a requisition message, its reverse path will be used for data forwarding. Otherwise, the data will be forwarded by flooding, reaching the sink anyway, and the path for the source nodes will be built on the next requisition period.

### 3.3. Estimation Model and Adaptive Rule

In this section, we describe how adaptation is performed, with the routing mechanisms previously described, to save energy.

In event-driven scenarios, a sensor node detects an event when its measured value represents a situation of interest. Once this occurs, we assume that the sensor node starts to generate and send data towards the sink.

**Figure 5.**
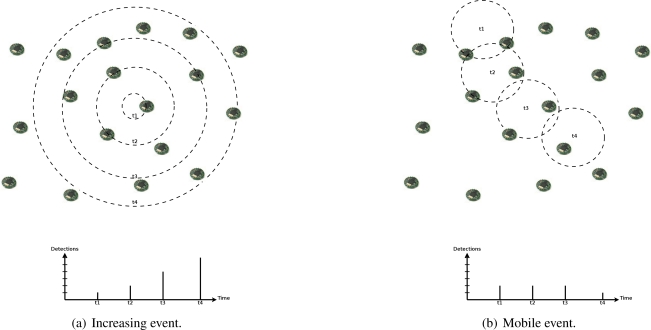
Examples of event occurrences and detections.

In such scenarios, we also expect spatial and temporal correlation in event detections by sensor nodes. In addition to the influence area of events, which causes simultaneous detections in a nearby area, events may present a seasonal characteristic, where they can be distributed in time following an occurrence rate, or a dynamic characteristic, where they can present an increasing actuation range or mobility (as the examples in Figure 5). Thus, we can expect that an event with certain characteristic will not change abruptly. For example, a mobile or increasing event will be detected by new nodes following its velocity or increasing rate, or a seasonal occurrence characteristic will be maintained, and its detection rate as well.

As described before, reactive algorithms are preferable if the network is inactive or events are rare. By analyzing the reactive and proactive algorithms presented before, we can observe that the cost of data flooding for path discovery in SID is similar to the cost of a tree-building in EF-Tree, which establishes a routing infrastructure for all reachable nodes at once. Thus, if we expect to have at least one source detecting events in the next time interval of route validity, the proactive behavior can be taken to avoid new discovery flooding and save energy. This knowledge is used by the adaptive model of Multi-MAF.

For event-detection estimation, we apply the simple signal processing method of Moving Average Filter (MAF) [25] on the number of new node detections (nodes starting to send data of an event). As the name suggests, this filter computes the arithmetic mean of a number of input measures to produce each point of the output signal, and this can be translated in the following equation:

(4) $$\mathrm{MAFout}[i]=\frac{1}{m}\sum_{j=0}^{m-1}\mathrm{input}[i+j],$$

where *m* is the filter’s window (number of input observations to be considered).

We have chosen the MAF method due to its capacity to capture the behavior of the last *m* intervals, which is good for seasonal occurrence detection, and due to its characteristic of step response, which is good for identifying any increase in correlated detections. It also filters the associated noise (caused by packet losses, queue delays, or an occasional event distribution) and improves the signal estimation. Other data fusion methods can be used for the node detection estimation [20], but MAF is simple and has low computational cost, necessary for constrained WSNs.

For the Multi-MAF’s adaptation, as soon as a high traffic condition is detected and a proactive behavior is assumed, more advantages can be achieved. Thus, we configured the monitoring periodicity with the same value of the data generation rate of the source nodes. The output of MAF is used as the estimate for the next period of observation. For example, if the monitoring period is 10s (used later in our simulations), the MAF results on the expected number of detections for the next 10s. As described before, we want the estimate for the next route validity interval, and by assuming this value as ten times the data rate (also used in our simulations), we can assume that if 10× *MAFout* ≥ 1 the proactive behavior will be taken. In other words, if at least a path discovery is expected for the next route validity interval (the cost of a flooding), it would be better to build the routing infra-structure for all the network in advance.

**Figure 6.**
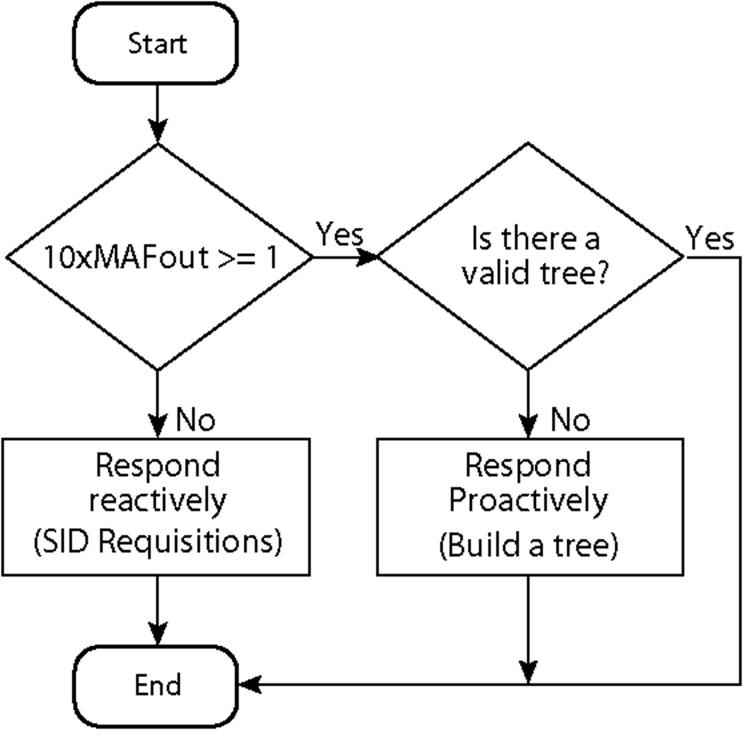
Multi-MAF’s adaptive rule.

If Multi-MAF is already operating in the proactive mode with a valid routing infrastructure, this action is not performed until the validity expires. The resulting adaptive rule of Multi-MAF is depicted in Figure 6.

## 4. Simulation and Evaluation

In this section, the algorithms described in this work are evaluated through simulation. We first present an evaluation of the *MAF* estimator to setup its parameters. Then, we describe some specific scenarios assessing the performance of the Multi-MAF implementation described in this work. We compare Multi-MAF with SID and EF-Tree alone, with other well-know algorithms in the literature, namely Push Diffusion and One-Phase-Pull Diffusion, and with the original version presented in [8].

### 4.1. Parameters

The experiments were performed with the ns-2 Network Simulator (http://www.isi.edu/nsnam/ns/). The simulation parameters were based on the Mica2 Sensor Node (http://www.xbow.com/): transmission power of 45.0 mW, reception power of 24.0 mW, bandwidth of 19200 bits/s, and a communication radius of 40 m. As this platform uses a CSMA/CA like MAC layer protocol, we used the IEEE 802.11 implementation available in ns-2 in ad hoc mode with the frame structure necessary for the routing protocols, i.e., we only used the RTS/CTS channel access scheme of the implementation with collision treatment. In fact, we cannot ignore the power consumption relative to the channel listening (corresponding to idle power), which is close to the reception power in the Mica2 nodes. But this consumption is much reduced in solutions like B-MAC [24] due to periodical wake-ups. Also, as it is the same for all the evaluated algorithms, which results in a constant amount of energy added to all algorithms (verified through simulations), we do not considered it for the comparative analysis.

In all simulations, we considered a 50-node network randomly distributed in an area of 100 × 100 m^2^ and only one sink. Both data and control messages have 20 bytes and are transmitted every 10 s and 100 s, respectively. The main metric evaluated was the energy consumption. All experiments were executed 33 times with a confidence interval of 95% (vertical bars in graphics).

### 4.2. MAF Estimator Evaluation

Due to the absence of a generic event model for WSNs, we represent the event-driven scenario by two types of detection distribution: Uniform and Gaussian distributions. In the first case, the variation on the detection ratio is represented by different number of sources generating data randomly during the simulation time. This distribution can represent the occurrence of uncorrelated event detections. In the second case, detections are distributed with a standard deviation around an average simulation time, and this distribution can represent the occurrence of correlated detections.

**Figure 7.**
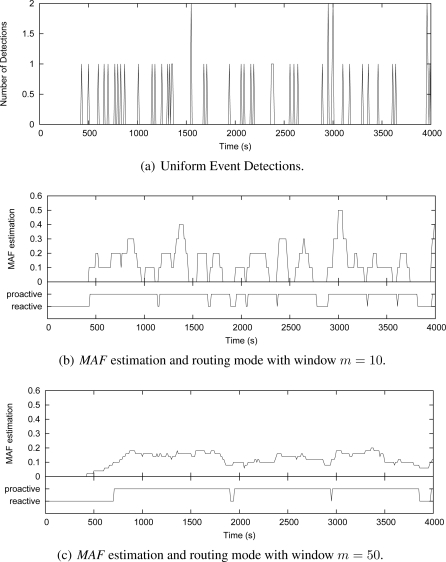
Multi-MAF’s adaptive model (uniform event detection).

**Figure 8.**
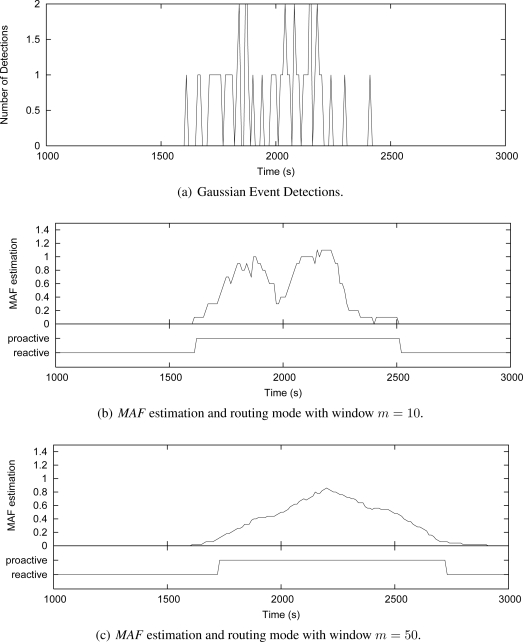
Multi-MAF’s adaptive model (Gaussian event detection).

To better understand how the *MAF* estimator works and how the window size *m* affects its performance, we show in Figures 7 and 8 some simulation cases that consider the Uniform and Gaussian distributions, 50 source nodes and window sizes of 10 and 50. These graphics show instances of nodes detecting events along the simulation time. The number of detections is measured every 10 s [Figures 7(a) and 8(a)]. For each instance, the grapthics present the *MAF* estimation, and the resulting operating mode of Multi-MAF (reactive or proactive mode) according with this MAF value.

Figure 7(b) shows that when *m* = 10, *MAF* does not properly capture the seasonality of detection occurrence in a Uniform distribution, causing several changes between the reactive and proactive modes. This behavior is clear reactive mode is set around 1100 s, due to an occasional empty interval, which do not reflect the overall behavior of detections. In the sequence, a new detection happens, which represents an additional cost, and Multi-MAF turns to proactive mode again. When *m* = 50 [Figure 7(c)], these transitions are not so frequent, and *MAF* has a better performance. However, in this case, *MAF* is slower to detect that the initial condition (initial occurrences around 500 s) has changed. The impact of *m* is also observed with Gaussian distribution in Figure 8(b),8(c). In this case, a lower *m* is better for responding faster to increases in the detections.

In all cases, the lower the filter window *m*, the faster the variation detection. On the other hand, the greater the filter window *m*, the smoother the estimation. Based on more simulation cases with the adopted parameters, we obtained *m* = 20 as the better value to capture seasonal characteristics of uniform detections, which does not respond so slowly to variations of the Gaussian distribution. This value represents an detection estimation based on two infrastructure building intervals (200 s) and it was used in the simulations of Multi-MAF presented below.

### 4.3. Multi-MAF Evaluation

#### Correlated detections

In the first scenario, we have sources generating random data during the time, according to a Gaussian distribution that represents the occurrence of correlated detections of events. We set the average of this distribution in the middle of the simulation time of 4000 s and standard deviation of 100 s. The duration of the data generation was a random value between 1 s and 100 s.

**Figure 9.**
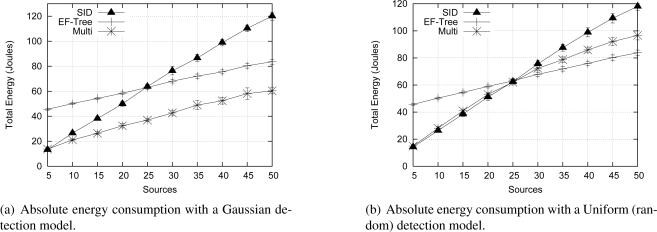
Performance of Multi-MAF under different traffic characteristics.

Figure 9(a) shows the summary of the consumed energy in the entire network after the specified simulation time, considering that the number of source nodes detecting an event varied from 5 to 50 nodes, randomly chosen. In this simulation, with a not very intense traffic, all algorithms delivered nearly 100% of the packets (this graphic was omitted). Regarding the energy consumed, Multi-MAF outperforms the other algorithms independent of the number of sources. With the lowest number of sources (5 sources) Multi-MAF and SID consume almost the same energy, however, the difference between them becomes close to 100% with 50 sources. The advantage of Multi-MAF is the adoption of the reactive behavior when the network is inactive, corresponding to energy savings related to EF-Tree, and the flooding avoidance of new sources when the proactive behavior is assumed, which represents a high cost to SID as the number of sources increases. This proactive behavior is taken when the *MAF* detects an increase in the traffic, so its value becomes higher than the threshold 0.1.

#### Uncorrelated detections

In the second scenario, based on the previous one, we represent the detection of uncorrelated events. The variation on detection rate is represented by different number of sources generating data randomly distributed over the simulation time of 4000 s according to a Uniform distribution.

In Figure 9(b), SID and Multi-MAF outperform EF-Tree when the number of sources is small (less than 25). In this case, Multi-MAF operates just like SID, due to a low number of detections by Multi-MAF’s *MAF* and consequently no proactive behavior is taken. This advantage happens because, with this reactive behavior, the routing infrastructure is created and maintained only when necessary and constant proactive behavior is unnecessary with a low amount of detections. However, when the traffic increases, EF-Tree begins to be more suitable, because it builds the routing infra-structure for all nodes at once and this proactive behavior is compensated by initial floodings of SID for path discovery. In this case, Multi-MAF’s performance becomes closer to the EF-Tree, outperforming SID. The reason is that MAF detects the high traffic condition and the proactive behavior is chosen. Again, since all algorithms present a high packet delivery ratio, this graphic was omitted.

In the last case, with high number of detections, Multi-MAF and EF-Tree performances are not the same, because since the simulation time is kept constant, the increasing number of sources leads to a decreasing inactivity time, amortizing the cost of the EF-Tree from the start of simulation. Since Multi-MAF always starts to operate as SID, changing to EF-Tree only when the MAF detects this traffic condition leads to initial data flooding. In a real event-driven scenario, we can expect to have longer inactivity periods than in the previous simulated scenario. Thus, the energy cost of the EF-Tree shifts to a higher value due to its proactive characteristic which results in a better relative performance for both SID and, in special, Multi-MAF.

#### Robustness

**Figure 10.**
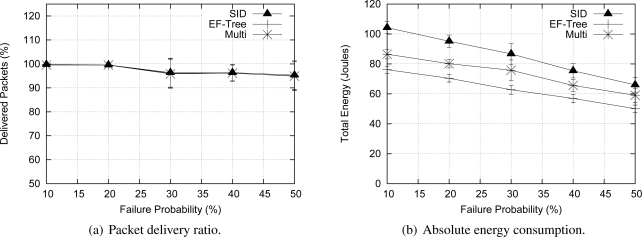
Multi-MAF’s performance under different probabilities of failure.

To assess the resilience of Multi-MAF, we fixed the number of sources in 50 nodes with a Uniform distribution in the simulation time, and varied the probability of node failures from 0% to 50% randomly during the simulation. When a node fails, it stays inactive until the end of simulation. Figure 10(a) shows that all algorithms presented a similar performance regarding the delivery ratio (relation between received and data packet sent), because in all cases the time to recover from a failure was the same (10 times the data rate). Regarding the energy consumption [Figure 10(b)], EF-Tree outperforms SID. The reason is that in the SID algorithm, sources have to flood messages for path discovery, whereas the EF-Tree algorithm rebuilds the tree for the entire network at once. Multi-MAF’s average performance stayed between EF-Tree and SID performances, because it can operate in one of these two modes during the network lifetime. As we can see, the results present large confidence intervals. This is due to the random node failures occurring at important locations of the network with a high effect on the overall data delivery ratio and energy consumption.

### 4.4. Comparison with other algorithms

**Figure 11.**
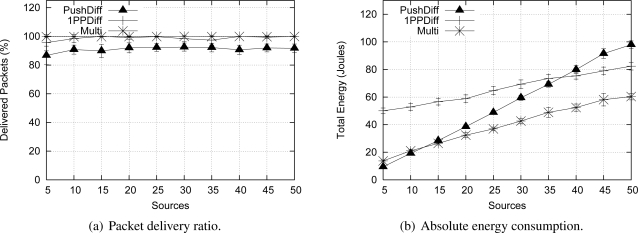
Comparison with Diffusion versions in the correlated detection scenario.

**Figure 12.**
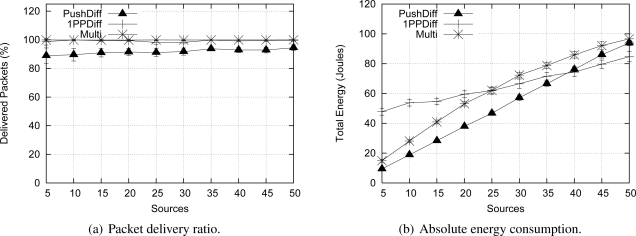
Comparison with Diffusion versions in the uncorrelated detection scenario.

In this section, we compare Multi-MAF to other algorithms, namely the Push Diffusion and One-Phase-Pull (1PP) Diffusion algorithms [13]. We use the same scenarios described before, and set Push and 1PP Diffusion parameters—such as data rate, control messages (interests and reinforcements) periods and packet sizes—to be fair with the Multi-MAF parameters. The simulation results are presented in Figures 11 and 12.

Figure 11 shows that Push Diffusion and 1PP Diffusion behave similarly SID and EF-Tree, respectively. A wider comparison among these protocols is performed by Figueiredo *et al.* [26]. The reason is that Push Diffusion and SID are similar proposals, infrastructure construction and maintenance; and so are 1PP Diffusion and EF-Tree. The main difference between SID and Push Diffusion is that in Push Diffusion, exploratory packets are sent in longer fixed periods, while in SID data are broadcast until paths are defined. This difference makes SID more appropriated for event-driven scenarios and Push Diffusion more vulnerable to eventual packet losses, as the delivery ratio graphics show. Obviously, when fewer packets are delivered, the absolute energy consumption is lower, which is not an advantage in this case. In summary, compared to these classical solutions, Multi-MAF achieves some benefits, since it adapts to different dynamic conditions.

**Figure 13.**
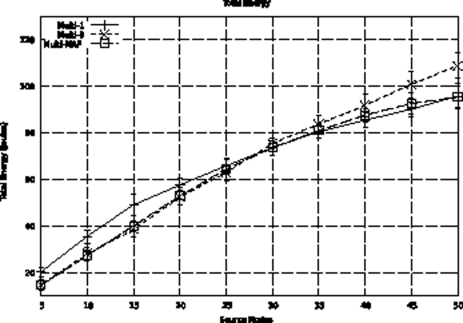
Comparison with threshold version.

The previous hybrid adaptive proposal [8] uses a threshold for the number of sources as an adaptation model. This model does not properly represent the event occurrence characteristics. For example, occasional distribution of event detections can lead to a concentrated measurement higher than the established threshold. In this case, a proactive behavior that may not correspond to event occurrences, which are better captured using MAF. We also compared the Multi-MAF with the original version in the Uniform detection scenario, which better presents the variance in detections. We use thresholds of 1 (Multi-1) and 3 (Multi-3) for Multi, because they lead to the best performance in the lower and high traffic cases, respectively. As we can see in Figure 13, Multi-MAF results, with the new adaptive model, are closer to the best performance of the other algorithms.

## 5. Conclusions and Future Work

In this work, we proposed an adaptive hybrid approach for routing in WSNs, called Multi-MAF. Multi-MAF aims at autonomously adapting the routing strategy to improve the energy efficiency, when the network presents different traffic profiles. An event detection model was proposed to support the adaptive decision behind Multi-MAF. As a result, we showed that when operating conditions of the network are unpredictable, adaptive approaches, such as Multi-MAF, are more suitable than a fixed routing strategy, leading to resource savings.

To the best of our knowledge, there is no other solution for WSNs considering hybrid adaptive behavior for routing. Although this approach was first introduced in the MANET domain (see Section 2), this work differs from those, since we consider the traffic characteristic of WSNs to estimate event detections, and reduce the impact of reactive path discovery by choosing a proactive strategy. This is an interesting approach for resource-constrained networks operating with dynamic traffic profiles. In addition, Multi-MAF considers a simple reactive and a simple proactive routing solution for WSNs.

As future work, we intend to improve both the reactive and proactive components of Multi-MAF. Recent contributions—such as BVR [14], VRR [15] and S4 [16]—present some optimizations to reduce the cost of routing updates. Consequently, these solutions are good candidates for composing the proactive component of Multi-MAF. Regarding the reactive component, the LRDE [19] solution proposes a heuristic to reduce the energy cost of path discovery. Thus, it could also be used as the reactive component of Multi-MAF. In any case, the adaptive model of Multi-MAF should be adjusted to determine the better value of event-detection estimation for changing the routing strategy. We intend to evaluate other techniques for event estimation, and evaluate the impact on our strategy when we have other functions, such as data aggregation and node scheduling. We also plan to implement and evaluate this solution in real sensor platforms and scenarios. The proposed approach can be used to the construction of new hybrid adaptive solutions that include other classes of routing algorithms, for instance, geographical and hierarchical algorithms.
